# Tacrolimus—why pharmacokinetics matter in the clinic

**DOI:** 10.3389/frtra.2023.1160752

**Published:** 2023-08-21

**Authors:** Lino Henkel, Ulrich Jehn, Gerold Thölking, Stefan Reuter

**Affiliations:** ^1^ Department of Medicine D, University of Münster, Münster, Germany; ^2^ Department of Internal Medicine and Nephrology, University Hospital of Münster Marienhospital Steinfurt, Steinfurt, Germany

**Keywords:** tacrolimus, tacrolimus pharmacokinetics, tacrolimus formulation, tacrolimus metabolism, kidney transplantation, renal transplantation

## Abstract

The calcineurin inhibitor (CNI) Tacrolimus (Tac) is the most prescribed immunosuppressant drug after solid organ transplantation. After renal transplantation (RTx) approximately 95% of recipients are discharged with a Tac-based immunosuppressive regime. Despite the high immunosuppressive efficacy, its adverse effects, narrow therapeutic window and high intra- and interpatient variability (IPV) in pharmacokinetics require therapeutic drug monitoring (TDM), which makes treatment with Tac a major challenge for physicians. The C/D ratio (full blood trough level normalized by daily dose) is able to classify patients receiving Tac into two major metabolism groups, which were significantly associated with the clinical outcomes of patients after renal or liver transplantation. Therefore, the C/D ratio is a simple but effective tool to identify patients at risk of an unfavorable outcome. This review highlights the challenges of Tac-based immunosuppressive therapy faced by transplant physicians in their daily routine, the underlying causes and pharmacokinetics (including genetics, interactions, and differences between available Tac formulations), and the latest data on potential solutions to optimize treatment of high-risk patients.

## 1. Introduction

Currently, the calcineurin inhibitor (CNI) Tacrolimus (Tac) is the most prescribed immunosuppressant after renal transplantation (RTx), as approximately 95% of recipients are discharged with a Tac-based immunosuppressive regimen (1). It is recommended by the *The Kidney Disease: Improving Global Outcomes* (KDIGO) guideline as the main cornerstone immunosuppressant after RTx (2) because it offers a significant reduction in acute rejection rates as well as an increase in long-term allograft survival (3). Despite being highly effective, its numerous adverse effects such as nephrotoxicity, infection rates, post-transplant diabetes, neurotoxicity, hypertension, and malignancies are limiting its usage (4). Therefore, many efforts have been made to develop alternative immunosuppressive regimens or improve drug galenic, especially for patients suffering from adverse effects or at risk of unfavorable outcome. Multiple studies on dose reduction by combining Tac with mechanistic target of rapamycin (mTor) inhibitors or belatacept, as well as elimination by switching to other immunosuppressant drugs have been published lately (5, 6). Therein, everolimus-based immunosuppression showed comparable efficacy in combination with (reduced) Tac compared with a standard regimen in renal and liver transplant patients, with comparable increases in renal function and treatment failure rates, but lower cytomegalo- and BK polyomavirus infection rates (7–9), most likely by retaining CMV-specific T-cell functionality (10), leaving both protocols safe and effective (11).

Besides the adverse effects, the high inter- and intraindividual variability (IPV) in pharmacokinetics (PK) and -dynamics pose a major challenge to physicians at reaching a given target trough level within the narrow therapeutic window. While therapeutic drug monitoring (TDM) is essential to prevent underexposure associated with increased rejection risk, and overexposure, which leads to an increase in adverse events, it has limitations in predicting the required Tac dose, due to the many factors that impact the Tac metabolism (12). These include external influencing factors such as drugs, foods, and herbs along with internal aspects like gastrointestinal factors as well as sex, age, serum albumin, hematocrit, and genetic variants, i.e., in the cytochrome P450 (CYP3A4/5) expression. Beside of that, the individual response of patients to Tac treatment and the different galenics of the available Tac formulations lead to distinct pharmacokinetic profiles, which can result in over- or underexposure, although the measured trough concentrations are always within the therapeutic window (13).

Despite the aforementioned potentially toxic profile and limitations of TDM, Tac remains the most relevant immunosuppressant in therapy of patients after solid organ transplantation, which has led to a broad variety of studies addressing Tac metabolism and optimization of dosing. Even with machine-learning based approaches, statistic modelling, and genetic analyses, which have been proven to be effective in dose calculation (14–16), a simple and cost-effective tool for clinical practice is not yet available.

With this review, we want to offer an update on the current knowledge of Tac pharmacokinetics and provide assistance to physicians in clinical practice by presenting a simple and cost-effective tool, the C/D ratio, which can support the management of Tac-based immunosuppressive therapy.

## 2. Factors influencing Tac pharmacokinetics

### 2.1. CYP3A4, CYP3A5 and P-glycoprotein

One of the most important determinants of Tac PK is the first-pass effect, regulated by Cytochrome P450 enzymes CYP3A4 and CYP3A5 as well as the P-glycoprotein (P-gp), which are predominantly expressed in the liver and gut, but can also been found in other tissues. Their function depends on genetic polymorphisms (12). It is important to note that CYP3A4/5 and P-gp are involved in the metabolization process of a wide variety of drugs and susceptible to induction or inhibition (17, 18).

P-gp is an ATP-dependent efflux pump located in the cell membrane that, among other drugs, transports Tac back into the intestinal lumen. CYP3A4 and CYP3A5 are monooxygenases and involved in the so-called phase I metabolism by catalyzing many reactions of multiple different drugs (19, 20). Phase II metabolism on the other hand occurs exclusively in the liver by glucuronidation, conjugation, acetylation, sulfation, and demethylation. After phase I and phase II, Tac is metabolized into at least 8 different products (21), potentially involved in mechanisms of Tac toxicity (22). Some metabolites are described in literature, such as M-I, M-II and M-III which are mono-demethylated, M-V/VI/VII which are di-demethylated, the mono-hydroxylated M-IV and M-VIII, which is modified by multiple reactions (23, 24). Despite being known for some years, data on this subject are inconsistent, and the pharmacological activity of Tac metabolites requires further investigation. Overall, Tac is largely metabolized by the liver and excreted via the bile (∼97%) and urine (2%), with less than 1% of the compound remaining unmetabolized in urine and feces (25).

### 2.2. Gastrointestinal parameters

After oral Tac intake, it undergoes presystemic metabolism, which mainly involves CYP3A4 and CYP3A5 metabolism in gut and liver as well as P-gp transport into the intestinal lumen (26, 27). When Tac enters an enterocyte of the intestinal mucosa as a highly lipophilic substance, it either migrates to the basolateral side and into the blood or is eliminated by CYP3A enzymes and P-gp. The latter keeps the intracellular Tac concentration low and therefore prevents CYP3A from saturation (28–30). Elimination of Tac metabolites occurs primarily through biliary excretion (more than 95%), while only 0.5% of the parent drug could be found unchanged in the urine or feces (30). On top of that, Guo et al. recently found an additional elimination route in which commensal gut bacteria (mainly Clostridiales) convert Tac into the 15-fold less potent metabolite M-I (31).

Because of several gastrointestinal parameters, absorption and bioavailability of Tac (when administered orally) are highly variable (32), leading to the high IPV mentioned earlier. First of all, gastric pH and motility impact Tac absorption directly (33). The latter could be an explanation for the circadian and time-dependent changes in Tac PK, as studies showed a significantly lower Tac peak level (Cmax) and area under the curve (AUC) after nighttime administration than after morning administration, while it is known that the gastric emptying rate physiologically decreases in the evening time (13, 34, 35). This is supported by the fact that CYP3A expression decreases in the distant intestine and an influence of the CYP3A expressor status on Tac chronopharmacokinetics could not be found (36). In addition, the Tac level appears to be relatively constant during the day and night when using continuous i.v. application, as described in a small number of 10 patients (37). Interestingly, despite the lower measured AUC after nocturnal dosing, a recent study showed no significant reduction in pharmacodynamic effect (38).

Tac bioavailability is generally influenced by food intake. In particular, high-fat content food reduces Tac absorption in the gut, which is why it is recommended to eat 2 h before or 1 h after taking Tac (39, 40). In addition to that, several food components, dietary supplements and herbs can have an influence on Tac metabolization, i.e., an inhibition of CYP3A4 by grapefruit juice should be noticed (41, 42).

As mentioned before, the intestinal first-pass mechanism is highly responsible for the IPV after Tac exposure, with the hepatic metabolic component likely predominating (43). One explanation could be the different distribution of CYP3A and P-gp within the intestine, as CYP3A expression decreases more distally and P-gp expression, though having a very high variability, tends to increase and reaches its maximum in the colon (44–46). Analogous to the decrease of CYP3A expression, Tac absorption mainly occurs in the distal part of the intestine despite the higher P-gp concentration. This leads to higher Tac absorption rates with decreased intestinal transit time as the Tac concentration increases in the distal intestine (47, 48). Which again could be an explanation for the significant reduction of the necessary dose in patients receiving LCP-Tac, an extended-release formulation, in comparison to an immediate release Tac intake (13).

It is well known that patients suffering from diarrhea show increased Tac trough levels. This can be partially explained by the aforementioned mechanism as the main absorption area shifts from the proximal to the distal intestine. Additionally, besides increased hemoconcentration and decreased hepatic blood flow (which negatively influences Tac metabolism), intestinal damage and inflammation could lead to increased drug permeability and negatively affect CYP3A and P-gp expression, particularly in the lower intestine (49, 50).

### 2.3. Genetic polymorphisms

As mentioned before, the high inter- and intrapatient variability of Tac PK poses a problem for clinical management, especially when defining the starting dose after transplantation as well as dose adjustment of ongoing therapy. While this can partially be attributed to different genetic variations of participating factors in Tac metabolism such as CYP3A enzymes and membrane drug transporters, pharmacogenetics emerges as a possible tool to assist physicians in clinical practice (51, 52).

Nowadays it is evident that fast metabolizers mainly express CYP3A5*1, while CYP3A5*3 expressors have been identified as slow metabolizers (53). CYP3A4 activity, however, showed no correlation to Tac parent/metabolite ratios, confirming CYP3A5 as the single most important determinant of Tac metabolism (54). Nevertheless, CYP3A5 status was not shown to have a decisive influence on IPV (55, 56). Therefore, it is not surprising that efforts trying to integrate CYP3A5 status into Tac dosing considerations were often disappointing regarding safety or outcome (57–59). This could be due to the fact that CYP3A5 is only a part of a broad variety of factors that influence Tac metabolism (51). Recent studies showed that approximately 16.3% of Tac's biovariability is explained with CYP3A5 expression, 43.3% in combination with clinical factors such as red blood counts and albumin, and only 70% when they included a total of 44 gene variants (15, 60). The latter shows the complexity of this topic as high throughput genetic screening was necessary, which requires high effort in terms of cost and interpretation and is therefore not suited for daily clinical practice (15). At least an approach considering clinical and demographical factors (with age and body surface area being the most effective ones) next to CYP3A genotyping showed promising results regarding individualizing Tac dose early after transplantation (14), but further studying on this topic is needed.

To date there is no general recommendation regarding pre-emptive genotyping, even though doubling the first Tac dose after solid organ transplantation is recommend in known CYP3A5 expressors. This leaves TDM as the main tool for managing Tac dose adjustment in clinical practice, as there are no further recommendations regarding pharmacogenetic biomarkers to date (51).

### 2.4. Blood and tissue distribution

After transfer into blood, lipophilic Tac is mainly bound to red blood cells through the FK506 binding proteins (FKBP), leading to a 4-114-fold higher Tac concentration in full blood compared to plasma (61, 62). Despite poor correlation with AUC, TDM by measuring full blood concentration 12 (C12)- or 24 (C24) hours after administration is most commonly used and currently state of the art (51). Other assessment points with better correlation to AUC (especially for once-daily formulations) have been published, i.e., C6 as the most accurate single point or in combination with C2 as a two-point model for AUC0-12 (63) and C2 plus C10 for AUC0-24 (64), but have not yet gained acceptance in clinical practice, mainly due to practical reasons (65). Analytical methods for Tac measurement include either different immunoassays or liquid chromatography coupled to tandem mass spectrometry (LC-MS/MS). LC-MS/MS is the current gold standard due to its high sensitivity and specificity and its ability to quantify multiple compounds simultaneously, but its usage is limited by the need for specialized trained staff and by high efforts and costs. This carries the risk of handling errors and, in addition to high assay heterogeneity, leads to high inter-laboratory variation, making global standardization challenging (66–68). As a result, almost half of laboratories worldwide primarily use immunoassays that are produced as commercial kits (67). However, these are less accurate due to cross-reactivity of the immunoassay antibodies with Tac metabolites and are associated with higher result variability (66, 69). Therefore, current research is focused on the development of new fully automated LC-MS/MS instruments (70, 71).

Because Tac binding to red blood cells is temperature sensitive as well as nonlinear, and the uptake by red blood cells is strongly dependent on FKBP concentration, it is practically difficult to separate full blood from plasma Tac (72). Recently, Yoshikawa et al. also suggested FKBP as a potential biomarker for predicting Tac PK, because of its important role in the distribution of Tac in red blood cells (73). However, plasma Tac is predominantly bound to albumin, lipoproteins, α_1_-acid glycoprotein, and globulins, whereas the unbound fraction is mainly responsible for therapeutic effects (32). Only unbound Tac is cleared by the liver, but because it is only released slowly by red blood cells, hepatic clearance is comparatively low (74). Therefore, Tac is a low-clearance substance.

As expected, whole blood concentration of the clinically relevant unbound fraction is strongly dependent to hematocrit and albumin levels. Lower hematocrit and albumin levels mean less full blood concentration, since excess unbound Tac is quickly eliminated by the liver with no difference in therapeutic effect or toxicity. Therefore, Tac full blood concentration in patients with anemia or hypalbuminemia may be underestimated, which may result in higher toxicity after increasing the Tac dose to maintain target trough levels (12, 75–77). Sikma et al. recently confirmed this correlation in early-stage patients after thoracic organ transplantation and suggested measuring hematocrit-corrected whole blood levels or using i.v.-application, aiming for a lower therapeutic range (78, 79). However, it should be noted that i.v.-administration of tacrolimus should be dose-reduced and continuous over 24 h to avoid bolus-associated toxicity (25).

For its therapeutic effect, Tac binds to and forms a complex with FKBP12 in lymphocytes and inhibits the activity of calcineurin (73). Intracellular concentration of Tac in lymphocytes correlates with full blood concentration (albeit poorly) and is influenced by age, sex, albumin levels, hematocrit, transplant duration and P-gp expression while determining the degree of T-cell-activation. However, it was not found to correlate with genetic variation in CYP3A enzymes and clinical outcome such as rejection rates (80–82).

Tacrolimus is extensively distributed in the whole body but its distribution in solid organ tissue behaves similarly to lymphocytes and is dependent on P-gp expression and intracellular metabolism. Tac clearance is not affected by low-grade renal or liver dysfunction (83). Tac, however, impairs renal function directly in an acute and chronical manner through vasoconstriction and nephrotoxicity (84). Besides systemic Tac levels, recent studies also showed a significant involvement of intrarenal accumulation in CNI-induced nephrotoxicity. Tac accumulation in the allograft was shown to be associated with Tac dose, full blood concentration and, most notably, acute nephrotoxicity. An association with CYP3A5 or ABCB1 genetics could not be found (85, 86). In addition, we found that the development and extent of acute Tac-induced nephrotoxicity were related to peak drug concentration; higher peak levels were significantly more often associated with toxicity in histology than lower levels (87).

### 2.5. Drug-drug interactions

Besides Tac, cytochrome P450 enzymes CYP3A4/5 metabolize a broad spectrum of different drugs, which can also act as inducers or inhibitors and thus influence Tac metabolism (88–90). In addition, Tac competes with other drugs on binding capacities of P-gp. For example, various antibiotics (i.e., macrolide), calcium antagonists, protease-/kinase inhibitors [antivirals for HIV (e.g., booster), SARS-Cov2 (e.g., nirmatrelvir/ritonavir), or CMV (e.g., maribavir)], or antimycotics such as azoles can inhibit the activity of CYP3A enzymes (47, 91, 92). With regard to azoles, Huppertz et al. described only very small increases in Tac exposure when using LCP-Tac, a new once daily Tac formulation, after CYP3A inhibition with voriconazole, resulting in comparatively little effect on AUC and biovariability, in contrast to immediate-release Tac (IR-Tac), and elegantly demonstrating the influence of drug galenics and CYP3A5 distribution in the gut (93).

Inducers of CYP3A enzymes relevant to transplant physicians medicine are isoniazid, rifampicin, several anticonvulsive drugs and most importantly St. John's wort, a long time used herb for the treatment of depression, which is still commonly used (94). Because it depends on the regulation of certain nuclear receptors, induction of CYP3A and P-gp occurs with a delay (47). Importantly, steroids, which are part of many immunosuppressive regimes after solid organ transplantation, also induce Tac metabolism and increase Tac clearance (95, 96).

### 2.6. Age

The age dependence of Tac metabolism has been the subject of several studies. Pediatric patients require weight-adjusted and usually higher Tac doses compared to adults to reach a certain trough level (97, 98). This effect was attributed to increased hepatic clearance because liver size is relatively larger in children normalized to body weight, whereas no age-related difference was seen with a Tac dose normalized to body surface area (99).

As for elderly patients, things become more difficult as physicians are faced with more comorbidities and a higher potential of drug-drug interactions (polypharmacy) as well as a different immune response (immunosenescence) and susceptibility to adverse effects (99). With age, the immune system weakens, which leads to more infectious complications but also lower rejection rates. Consequently, it was shown *in vitro* and in a mice model, that a compromised CD4+ T cell response is related to augmented immunosuppressive capacities of Tac in elderly patients (100). This leads to the suggestion to aim at lower target trough level. In addition, it has been suggested that renal susceptibility to Tac increases with kidney age (84), and since older recipients tend to receive older grafts than younger ones, this must be taken into account. On the other hand, older kidneys are more immunogenic than younger ones (101).

Regarding Tac PK, studies showed contradictory results regarding an age dependency. Unfortunately, the number of elderly patients in these studies was mostly small, so their power with respect to this question is limited (102–104). In theory, an age-dependence of Tac PK would be logical, as a reduced splanchnic and hepatic blood flow, higher body fat, and changes in gastric pH in the elderly should have an influence on the distribution and clearance of Tac. This is supported by data from Jacobson et al., describing higher Tac trough levels with increasing age (105) and our data, since we found a higher rate of slow metabolizers in elderly than in younger patients (106). In addition to that, David-Neto et al. showed a lower Tac clearance and need for higher doses in elderly patients up to 6 months after transplantation (107). The fact that the once daily Tac formulation (LCP-Tac) results in lower treatment failure rates than twice-daily capsules (IR-Tac) in patients with age >65 years also supports an impact of age on Tac PK (108). Beside of that, safety and efficacy studies in this patient group are still lacking and need to be further investigated in light of the increasing number of elderly renal transplant recipients.

### 2.7. Sex and pregnancy

According to several studies, female patients require higher Tac doses (83, 109, 110) and have a greater risk of experiencing adverse effects (111). In pregnant women, it is necessary to increase the Tac dose (about 25%–50% by the end of the first trimester) to maintain a target trough level even though the effective drug concentration is most likely overestimated (112). This is due to an increased Tac metabolism from a higher CYP3A4 activity as well as an increase in plasma volume next to hypoalbuminemia and anaemia (77). In a review from Chandra et al., the author suggests to stay in the target trough level, albeit risking over-immunosuppression, and only reduce Tac dose if nephrotoxicity is suspected (113). Congenital malformations have not been associated to Tac exposure, which is most likely because of an active P-gp efflux transport towards the mother even though Tac crosses the placenta. Although this prevents the foetus from higher Tac exposure, physicians should be aware that nephrotoxicity and hyperkalaemia can occur in the foetus as well. Although it is very unlikely that Tac excretion in the breast milk leads to adverse effects in the child, it is generally not recommended according to the SmPC (25, 77).

### 2.8. Ethnicity

Several studies have reported that African Americans require higher Tac doses compared to Caucasians or Asians (96, 114, 115) mainly due to differences in intestinal P-gp and CYP3A variants (105, 116). A recent observational study showed an association of CYP3A5*3 with higher doses in all ancestries, but CYP3A5*6 and CYP3A5*7 were present only in African Americans (117). The influence of CYPs in this context was confirmed by a study comparing the administration of LCP-Tac and IR-Tac in African Americans and showing that PK was significantly less affected in the case of LCP-Tac than IR-Tac (118). Native Americans usually require lower Tac doses due to a decreased oral Tac clearance (119).

### 2.9. Time after transplantation

After transplantation, high doses of steroids are initially administered, which induces CYP3A4 activity, requiring higher doses of Tac to reach target levels. Then, steroids are reduced over time, resulting in increased absorption of Tac and decreased CYP3A activity (120). Thus, required Tac doses decrease over time after transplantation (“maturation”), so TDM should be executed in a higher frequency (104, 121), especially in CYP3A5 expressors. In addition to that, haematocrit and albumin levels increasing as well, which amplifies the aforementioned effects (120).

### 2.10. Tac formulation

After its introduction as Prograf® by Astellas in Japan in 1993, several generics of IR-Tac were produced when the patent expired in 2008, the first being tacrolimus from Sandoz in 2009. Bioequivalence between the generic or branded drugs and the original Prograf® has been shown in at least 10 publications, recently reviewed by Kocur et al. (122), with the exception of the granular formulation Modigraf®, which showed a 23% and 18% higher mean for Cmax and AUC, respectively (123).

**Figure 1:**
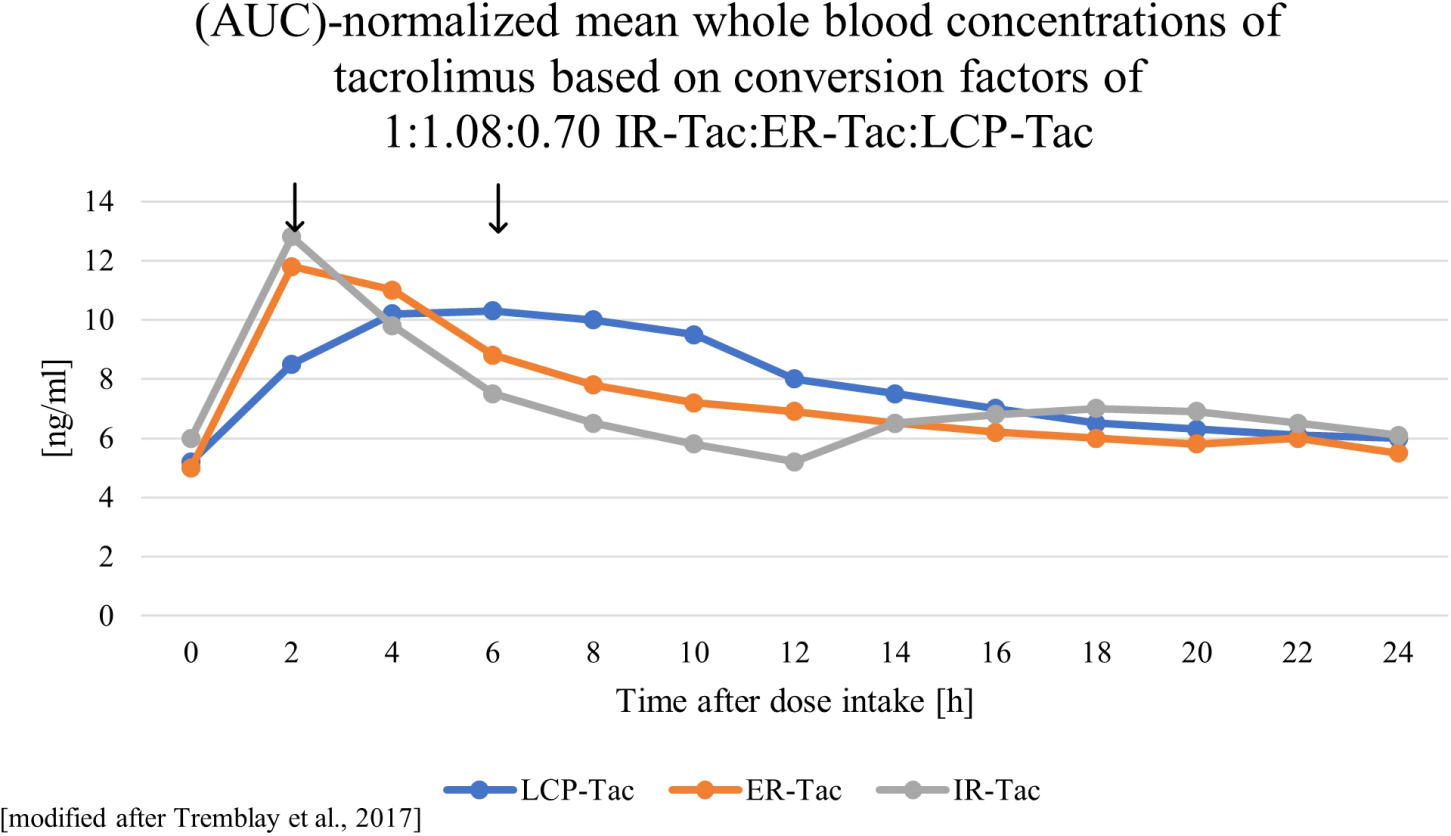
Schematic visualizations of the pharmacokinetics of different tacrolimus formulations. ER-Tac, extended-release tacrolimus, Advagraf®; IR-Tac, immediate-release tacrolimus, Prograf®; LCP-Tac, once-daily, Envarsus®, arrows mark the different peak concentrations.

As a reaction to the high variability in intrapatient trough levels, which are associated with worse outcomes (124–129), once-daily administered Tac formulations with prolonged drug release have been developed to improve bioavailability and adherence as well as lower intraday fluctuation and peak concentrations (Cmax), as illustrated in Figure 1. In addition to the widely established IR-Tac, granulate formulation extended release (ER)-Tac (Advagraf®) with a prolonged drug release (90% absorption after 6–12 h) and LCP-Tac (Envarsus®) with an additional improved bioavailability have been established in clinical practice, reviewed in detail by Piotti et al. (130). The latest formulation, LCP-Tac, uses MeltDose® technique, which improves solubility by breaking the particles down into the smallest possible units. This results in a progressive release of the drug in the distal intestine, which has much lower CYP3A activity and therefore lower Tac clearance (131). A recent randomized controlled trial compared the three Tac formulations in stable kidney transplanted patients and focused on PK (13). It showed a lower intraday fluctuation, a prolonged time (Tmax) to peak concentration (Cmax) and a significantly higher exposure on a per milligram basis for LCP-Tac than for ER- and IR-Tac. After exposure normalization, Cmax was noticeably lower for LCP-Tac vs. IR-/ER-Tac (roughly 17%), while Cmin only differed slightly to ER- (6% lower) and IR-Tac (3% higher). In accordance with other studies in liver and kidney transplant recipients (132, 133), a dose reduction of ∼30% after converting from IR- and 36% from ER- to LCP-Tac has been suggested. These different dose requirements also result in lower therapy costs (131, 134). In the ASTCOFF trial, ER-Tac did not show any differences in terms of exposure, Cmax, Tmax or fluctuation vs. IR-Tac, which is not always consistent with previous studies reporting slightly lower Cmax, longer Tmax and in some studies even a lower PK variability of ER-Tac compared to IR-Tac (135–137). The dose conversion rate from IR- to ER-Tac has been calculated to +8% (13). This means that higher doses are required (138), possibly attributed to lower saturation of CYP3A and P-gp by a prolonged release in the intestine and therefore resulting in an increase of the metabolism rate (47). Following the findings mentioned above, the recommended starting dose of IR-Tac with 0.1–0.3 mg/kg/day remained similar with ER-Tac. Because of lower dose requirements for reaching effective trough levels in *de novo* renal transplant recipients of 30%, as shown in a randomized controlled trial, the starting dose for LCP-Tac was reduced to 0.17 mg/kg/day (139). Interestingly, LCP-Tac was not able to significantly reduce IPV compared with IR-Tac or ER-Tac (140). This might be related to the limited influence of CYP3A5 status on IPV mentioned earlier.

However, despite differences in PK between the Tac formulations, prospective trials showing superiority of LCP-Tac in terms of patient outcome remain rare. Recently, trials demonstrated similar clinical outcomes of recipients treated with LCP-Tac or other tacrolimus formulations during the first months following *de novo* kidney transplantation (141, 142). However, a benefit for LCP-Tac has been demonstrated in patients at high risk of treatment failure (108) and with Tac-induced tremor (131, 143). Since the tremor and also the nephrotoxicity of Tac is related with a certain degree to Cmax, LCP-Tac (with lower Cmax) might be beneficial in susceptible patients, especially rapid metabolizers (87, 144). Using retrospective data, we observed that switching to LCP-Tac was associated with a noticeable recovery of renal function in fast metabolizers (145). In liver transplant recipients, improvement in renal function was observed 12 months after conversion from standard-release Tac (IR- or ER-Tac) to LCP-Tac (146). Because non-adherence is significantly associated with higher rates of antibody-mediated rejection and graft failure (147), one of the main aims in the development of once-daily Tac formulations was the improvement of drug adherence, which has been demonstrated by several studies (148–150).

## 3. Relevance of current Tac dose finding models and the role of the C/D ratio

The high inter- and intrapatient variability in Tac PK poses a problem to physicians in predicting Tac exposure in their patients. Given its association with impaired long-term allograft outcome, recent efforts have been made to develop models to optimize individual immunosuppressive therapy. As previously described, genetic testing strategies were related to high efforts and costs and often disappointing regarding safety or outcome (57–59, 151–153). Population PK models for Tac dose prediction have been established but are complicated and need further research, especially regarding newer Tac formulations (154, 155).

**Figure 2:**
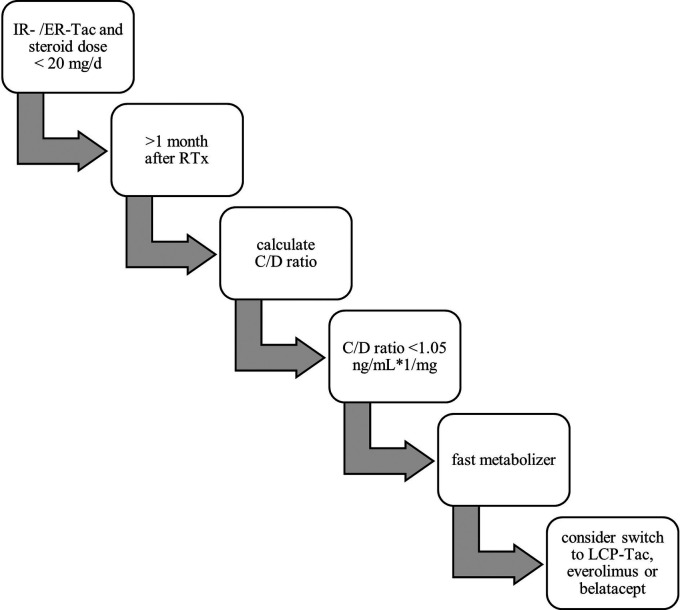
Flow chart for practical identification of fast metabolizers.

Despite the drawbacks such as high IPV and the narrow therapeutic window, Tac remains the most important immunosuppressant in patients after solid organ transplantation. To assist clinicians with a practical method for risk assessment rather than dose calculation, we previously proposed the calculation of the C/D ratio (106, 144). As presented in Figure 2 below, the C/D ratio is a simple and cost-effective tool that helps clinicians to optimize individual immunosuppressive therapy and identify patients at risk for adverse effects and unfavorable outcome.

The C/D ratio is calculated by dividing the Tac trough level, which is routinely measured, through the daily Tac dose at a specific time (usually later than 1 months after surgery) in the steady state after solid organ transplantation (106, 156). It should be noted that the C/D ratio can be calculated for every Tac formulation but cut-offs for definition of different metabolism groups are different. The performance of the C/D ratio has been extensively tested by our group and others in renal transplant patients. However, it should be noted that although there are many studies from different continents and transplant programmes with mostly consistent results, they are based on retrospective evaluations. A prospective study of liver transplant patients is currently recruiting in Germany, and several studies of kidney transplant patients are underway. Herein, we previously found a C/D ratio of 1.05 ng/ml × 1/mg to be a suitable cut off for distinguishing slow metabolizers (C/D ratio ≥1.05 ng/ml × 1/mg) from fast metabolizers (C/D ratio <1.05 ng/ml × 1/mg) when using IR-Tac. We demonstrated that fast metabolizers needed more indication biopsies, were more likely to experience biopsy proven CNI nephrotoxicity and BK virus infection, as well as to develop lower estimated glomerular filtration rate (eGFR) values during a 24-month follow-up after renal transplantation (106, 157). Following studies by our center showed that a low C/D ratio increases the risk of developing acute CNI-induced nephrotoxicity and was associated with a faster decline of eGFR, higher rejection rates and, most importantly, a reduced patient as well as overall graft survival within 5 years after RTx (87, 144). This was confirmed in 2020 by the TOMATO study, in which the authors described the C/D ratio as a predictor of death-censored graft survival (158). Interestingly, the French study included patients on IR-Tac and ER-Tac in their analysis. Recently we were able to demonstrate that the C/D ratio is a valuable tool in kidney transplanted patients treated with ER-Tac, showing that the concept of the C/D ratio works for different formulations (159). A recent Korean study also showed that a high C/D ratio correlates with lower delayed graft function rates, less acute rejection lower and increased eGFR 6 months after RTx (160). An association of high Tac clearance with the development of interstitial fibrosis and tubular atrophy (IFTA) was shown by Egeland et al. in 2019 and confirmed to be true for a low C/D ratio in a study 2 years later (161–163). However, we found no differences between slow and fast metabolizers in the incidence of diabetes mellitus, urinary tract infections, and dyslipidemia after transplantation (164–166).

As mentioned above, the results of prospective studies are still lacking. These are needed to make a definitive assessment of the appropriateness of the C/D ratio. In particular, it needs to be clarified whether, and if so which, changes in treatment lead to better outcomes after a low C/D ratio has been detected in individual transplant recipients.

CYP3A5-positivity (mainly CYP3A5*1) is known to be mostly present in fast metabolizers and to be associated with a lower eGFR as well as significantly increased risk of Tac-induced nephrotoxicity (167, 168). Therefore, a correlation of CYP3A5*1 with a low C/D ratio has been expected. Recent studies gained evidence of this assumption and found a strong association of CYP3A5 genotype with a low C/D ratio and a decline in eGFR values (60, 169, 170).

With growing evidence that calculation of the C/D ratio can identify Tac-fast metabolizers and therefore at-risk patients, the timing for assessment remains to be discussed. In a study from our center, patients with a Tac C/D ratio <1.05 ng/ml × 1/mg, calculated 3 months after renal transplantation, were characterized as fast metabolizers and had a significant reduced 5-year patient and overall graft survival, a faster decline in eGFR as well as higher rejection rates within 5 years after renal transplantation (144). An assessment 6 months after RTx also had good discriminative results for eGFR and acute rejection rates (160, 171). However, early assessment of the C/D ratio at day 0–7 did not predict results because too many influencing factors probably converge in the first days after transplantation (172). We conclude from our data that assessment of the C/D ratio should occur no earlier than 1 month, preferably 3 months, after transplantation, because by this time many highly variable factors such as hemoglobin, albumin, steroid dose have undergone some degree of stabilization and the data for the time points are compelling.

So how can the clinical physician optimize the immunosuppressive therapy after identifying a patient with a suspected unfavorable outcome because the patient is a fast metabolizer?

In the last years, we and others investigated this topic by modifying the immunosuppressive regimen either by changing the Tac formulation or switching the immunosuppressive regimen to everolimus or belatacept in selected patients. As fast metabolizers receiving ER-Tac also showed a decline in eGFR and increase of acute rejection rates similar to IR-Tac (159), patients with side effects were indication-based switched from IR- to LCP-Tac. In fast metabolizers LCP-Tac increased the bioavailability, the C/D ratio and was associated with a noticeable recovery of renal function while being safe (145). The same effect on C/D ratio was observed when African Americans, who have predominantly fast metabolism, were switched from IR-Tac to LCP-Tac (118). Conversion to everolimus led to a noticeable increase in renal function in both slow and fast metabolizer groups with a tendency towards a higher increase in fast Tac metabolizers (173). In addition, conversion to belatacept could also be an option, as an increase in eGFR was observed at 12 months post-transplant (174, 175). We hypothesize that the higher rate of adverse effect in fast metabolizers is caused by the higher daily doses required, which ultimately result in higher peak levels with comparable AUC and trough levels (87, 144, 176).

## 4. Conclusion

Despite its relevant side effects and narrow therapeutic window, Tac is the most commonly prescribed immunosuppressant after renal transplantation. Hence, regular TDM and dose adjustment is necessary, which poses difficulties for clinicians given the high PK variability. Therefore, it is relevant to physicians to know factors influencing its metabolism. A simple and effective tool, the C/D ratio, can classify patients receiving Tac into two major metabolism groups, and therefore helps to predict patientś risk for an unfavorable outcome. Based on this assessment, physicians could optimize and individualize immunosuppressive therapy, e.g., by switching to a different Tac formulation, everolimus or belatacept.
